# Contrast-enhanced CT findings-based model to predict MVI in patients with hepatocellular carcinoma

**DOI:** 10.1186/s12876-022-02586-2

**Published:** 2022-12-28

**Authors:** Qi Yue, Zheyu Zhou, Xudong Zhang, Xiaoliang Xu, Yang Liu, Kun Wang, Qiaoyu Liu, Jincheng Wang, Yu Zhao, Yin Yin

**Affiliations:** 1 Department of Hepatobiliary Surgery, The Affiliated Drum Tower Hospital of Nanjing University Medical School, Nanjing, China; 2 Department of Hepatobiliary Surgery, Nanjing Drum Tower Hospital Clinical College of Jiangsu University, Nanjing, China; 3 Department of General Surgery, Nanjing Drum Tower Hospital, Chinese Academy of Medical Science & Peking Union Medical College, Graduate School of Peking Union Medical College, Nanjing, China; 4 Department of Hepato-Biliary-Pancreatic Surgery, The Affiliated Changzhou No.2 People’s Hospital of Nanjing Medical University, Changzhou, China; 5 Department of Medical Imaging, School of Medical Imaging, Nanjing Medical University, Jiangning, Nanjing, China

**Keywords:** Hepatocellular carcinoma, Microvascular invasion, Nomogram, Preoperative prediction

## Abstract

**Background:**

Microvascular invasion (MVI) is important in early recurrence and leads to poor overall survival (OS) in hepatocellular carcinoma (HCC). A number of studies have reported independent risk factors for MVI. In this retrospective study, we designed to develop a preoperative model for predicting the presence of MVI in HCC patients to help surgeons in their surgical decision-making and improve patient management.

**Patients and Methods:**

We developed a predictive model based on a nomogram in a training cohort of 225 HCC patients. We analyzed patients’ clinical information, laboratory examinations, and imaging features from contrast-enhanced CT. Mann–Whitney U test and multiple logistic regression analysis were used to confirm independent risk factors and develop the predictive model. Internal and external validation was performed on 75 and 77 HCC patients, respectively. Moreover, the diagnostic performance of our model was evaluated using receiver operating characteristic (ROC) curves.

**Results:**

In the training cohort, maximum tumor diameter (> 50 mm), tumor margin, direct bilirubin (> 2.7 µmol/L), and AFP (> 360.7 ng/mL) were confirmed as independent risk factors for MVI. In the internal and external validation cohort, the developed nomogram model demonstrated good diagnostic ability for MVI with an area under the curve (AUC) of 0.723 and 0.829, respectively.

**Conclusion:**

Based on routine clinical examinations, which may be helpful for clinical decision-making, we have developed a nomogram model that can successfully assess the risk of MVI in HCC patients preoperatively. When predicting HCC patients with a high risk of MVI, the surgeons may perform an anatomical or wide-margin hepatectomy on the patient.

## Introduction

Liver cancer is the fifth most prevalent of all malignancies based on the latest data from the National Cancer Center, and hepatocellular carcinoma (HCC) is the most common type of liver cancer [1]. Currently, curative partial hepatectomy and liver transplantation are the primary treatment options for HCC patients. However, 40–70% of patients relapse within five years of resection, and 35% of patients relapse after liver transplantation [2, 3]. This is why its prognosis remains poor, despite liver cancer's decreasing incidence and death.

Unlike macrovascular invasion, which can be diagnosed by imaging, microvascular invasion (MVI) is a histological finding that can only be diagnosed pathologically after surgery [4]. In the presence of MVI, tumor cells can spread within the liver, eventually forming multiple lesions in the liver, portal vein cancer thrombosis, or distant metastases. So MVI is an independent risk factor for early recurrence and poor OS in HCC patients [5–7]. Zhao H et al. showed that HCC patients with MVI can benefit from anatomical liver resection in aspects of disease-free survival compared to non-anatomical liver resection [8]. Another study demonstrated that hepatic resection with wide margins (≥ 1 cm) is superior to narrow margins in patients with good liver reserve function, especially in patients with a high preoperative predicted risk of MVI [9]. In addition, after liver resection, HCC patients with MVI can benefit from adjuvant therapy with transarterial chemoembolization and oral sorafenib in aspects of OS, recurrence free survival or progression free survival [10–12]. As histopathological diagnosis has a limited impact on preoperative decision making, predicting the presence of MVI before surgery can help surgeons select the exact surgical approach then ultimately improve the prognosis of HCC patients.

Nomogram is a reliable tool for integrating and quantifying essential risk factors for disease prognosis [13]. A number of nomograms have been developed for MVI prediction in HCC patients [14–16]. For instance, Zhou Q et al. reported alkaline phosphatase (> 125U/L), alpha-fetoprotein (within 20–400 or ≥ 400 ng/mL), protein induced by vitamin K absence-II (within 40–400 or ≥ 400mAU/mL), tumor diameter, multiple tumors, pseudo-capsule, the infiltrative border with an irregular shape, and intratumor hemorrhage were independent risk factors of MVI [16]. While the nomogram developed by Mao S et al. identified only three independent risk factors for MVI (tumor diameter, serum AFP > 400 ng/mL, and total bilirubin > 23 µmol/L) [17].

Both contrast-enhanced CT and laboratory tests are an integral part of the routine management of HCC patients. So, it is meaningful to analyze these clinical examinations to obtain variables of predictive value. The purpose of our study is to develop a non-invasive predictive model for preoperative assessment of the risk of MVI in HCC patients based on the patient characteristics of our center.

## Methods

### Participants

In this study, we retrospectively analyzed the medical data of HCC patients who had partial hepatectomy or liver transplantation from two hospitals. We analyzed data on HCC patients from January 2017 to August 2020 at Nanjing Drum Tower Hospital and from January 2017 to June 2019 at The Affiliated Changzhou No.2 People's Hospital of Nanjing Medical University. The ethics committees of both hospitals have approved this study. Because of the retrospective nature of this study, the request for written informed consent from patients was waived.

Patients who met the below criteria were included: (a) contrast-enhanced CT imaging within one month before biopsy; (b) tumor specimens were histologically confirmed to be HCC with MVI; and (c) had partial hepatectomy or liver transplantation. The exclusion criteria were: (a) with other tumors; (b) incomplete clinical data; (c) contrast-enhanced CT imaging over one month before biopsy; (d) missing pathological data; (e) poor image quality; and (f) had received other treatments.

Clinical and pathological data were collected from medical records. Basic patient information included sex and age. Liver disease included hepatitis B virus (HBV) infection, hepatitis C virus (HCV) infection, and other. Laboratory measurements included aspartate aminotransferase (AST), alanine transferase (ALT), red blood cell (RBC), hemoglobin, platelet, total bilirubin (TBil), direct bilirubin (DBil), total protein, albumin, C-reactive protein (CRP), Albumin-Bilirubin (ALBI) grade, carbohydrate antigen 242 (CA242), carcinoembryonic antigen (CEA), and alpha-fetoprotein (AFP). Imaging features are described below.

**Fig. 1:**
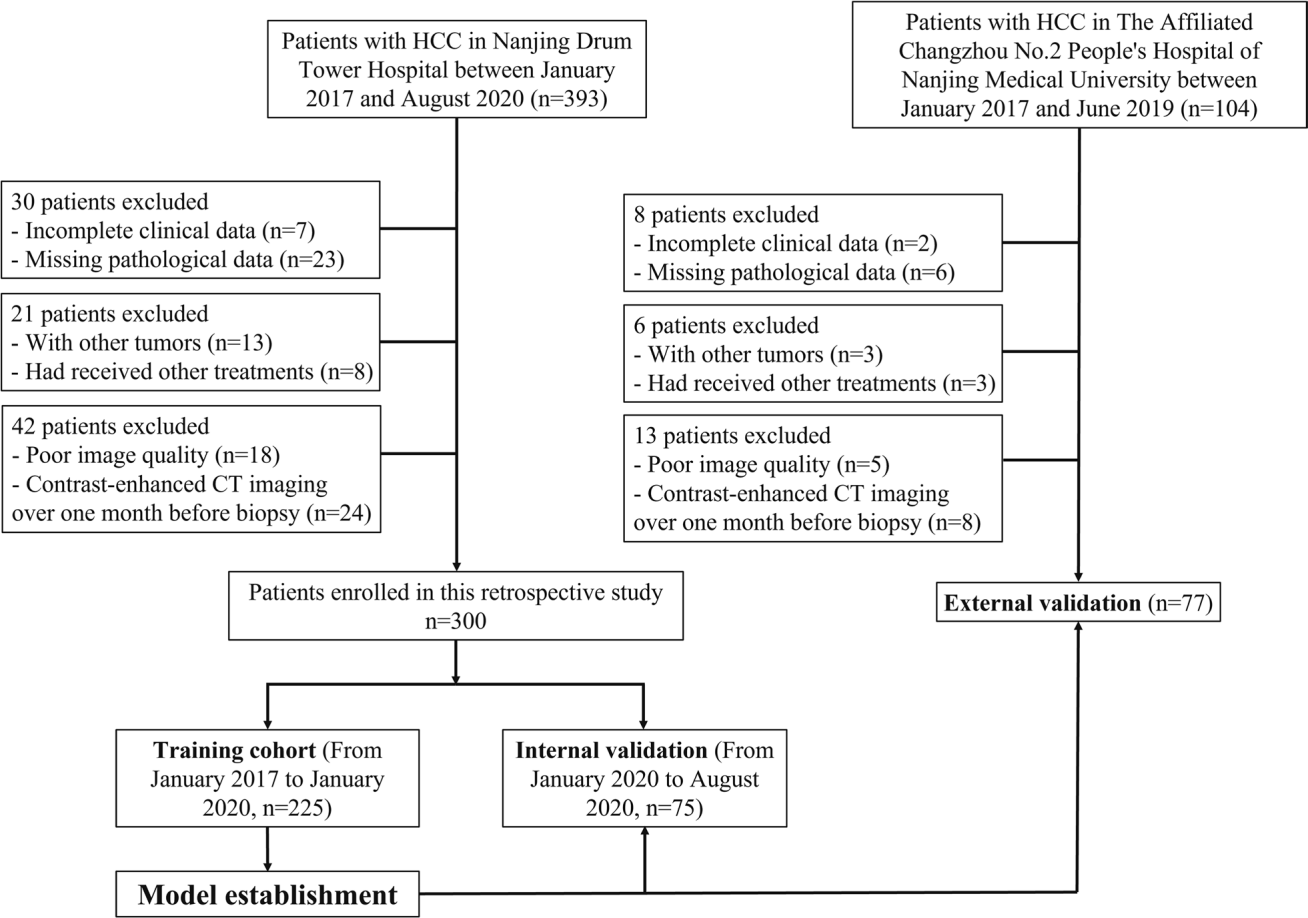
Patient selection flowchart. HCC, hepatocellular carcinoma; CT, computed tomography

The recommended criteria for our protocol requirements and contrast-enhanced CT are used according to the guidance of the American Association for the Study of Liver Diseases (AASLD) (Fig. 1).

### Contrast-enhanced CT technique and radiological evaluation

**Fig. 2:**
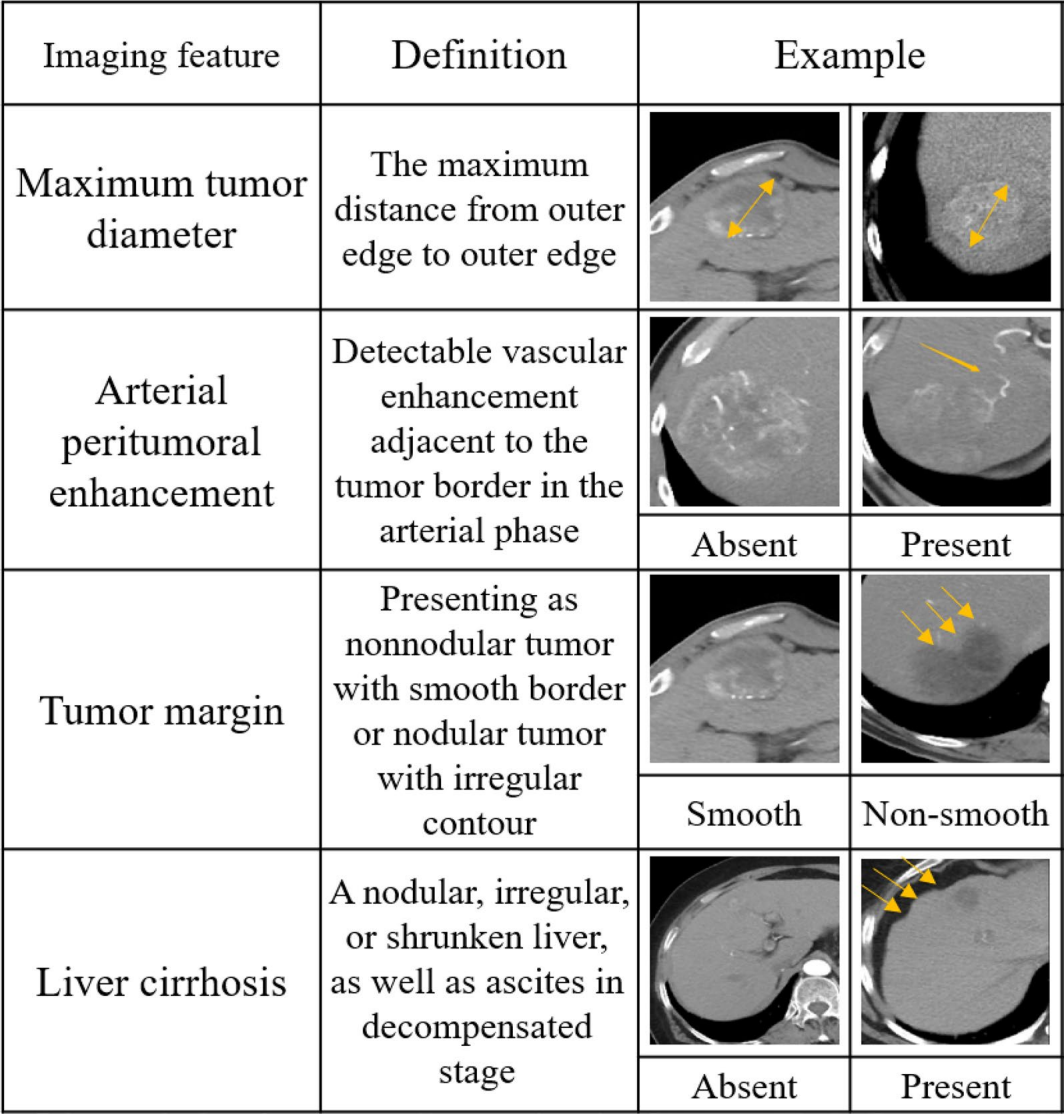
Definitions of major semantic features and representative CT images

Contrast-enhanced CT scans were obtained supine with 0.75–1.5-mm-thick sections and a 0.75–1.5-mm reconstruction interval. Iohexol (a contrast agent) was infused via peripheral vein before examinations. Two radiologists independently evaluated each patient's CT images, including the following features: (a) maximum tumor diameter; (b) tumor margin; (c) cirrhosis; (d) arterial peritumoral enhancement; and (e) tumor number. The diameters of the tumors were recorded as mean values (measured by two independent radiologists), and Fig. 2 shows the diagnostic criteria and representative CT images. We resolve any controversies in feature assessment through discussion.

### Histopathological assessment

Surgical specimens from each patient were examined independently by two pathologists for the presence of MVI, defined as a nesting mass of cancer cells seen microscopically in the lumen of the endothelium-lined vasculature. They were unaware of any patient's clinical features and imaging data and used hematoxylin–eosin staining and immunohistochemistry to confirm the pathological diagnosis.

### Model development and validation

In the training cohort, multiple logistic regression analysis was performed to build a model for predicting the presence of MVI in HCC patients. A nomogram was also constructed to provide a more coherent approach to risk assessment. The diagnostic performance of our model is evaluated in the internal and external validation cohorts using threshold values and weighted values formula obtained from the training cohort.

### Statistical analysis

The *χ*2 and Mann–Whitney U tests were used to compare categorical and continuous variables. These two tests were used to identify factors with significant differences between the MVI = 0 and MVI = 1 cohorts for follow-up multiple logistic regression analysis. Factors with a *p*-value < 0.05 were identified as independent predictors in the multiple logistic regression analysis. Nomogram was used to demonstrate the predictive model based on the *β* coefficients of each factor. *R* software (version 3.5.1) completed all statistical analyses. The prediction model's performance was assessed using ROC curves and the AUC. A *p*-value < 0.05 was thought to be statistically significant.

## Results

### Participants enrolled and baseline characteristics

393 and 104 HCC patients from Nanjing Drum Tower Hospital and The Affiliated Changzhou No.2 People's Hospital of Nanjing Medical University were included in the initial analysis, respectively. Figure 1 shows the flow chart for patient selection in detail. Finally, 225 of the 300 patients between January 2017 and January 2020 were distributed to the training cohort, and the remaining 75 patients between January 2020 and August 2020 were for internal validation. 77 patients were for external validation.

Table 1 shows the baseline characteristics of patients in the training and validation cohorts. There were no significant differences in age, gender, liver disease, imaging features, laboratory tests (including tumor markers), or the presence of MVI between these three groups. In the training and validation cohorts, the incidence of MVI in HCC patients was 29.3%, 38.7%, and 26%, respectively. These rates suggested that predicting MVI preoperatively is essential.

**Table 1:** Baseline characteristics

Characteristics	Training (*n* = 225)	Internal validation (*n* = 75)	External validation (*n* = 77)	*P*-value
Age in years^#^	58 (51–65)	59 (50–66.5)	60 (54–70)	.070
*Sex*				.833
No. of men	181 (80.4)	62 (82.7)	64 (83.1)	
No. of women	44 (19.6)	13 (17.3)	13 (16.9)	
*Liver disease*				.964
HBV infection	202 (89.8)	66 (88.0)	67 (87.0)	
HCV infection	9 (4.0)	4 (5.3)	4 (5.2)	
Other	14 (6.2)	5 (6.7)	6 (7.8)	
Maximum diameter^#^	46 (28–72)	49 (34–64)	41 (26–65)	.652
*Tumor margin*				.060
Non-smooth	109 (48.4)	46 (61.3)	33 (42.9)	
Smooth	116 (51.6)	29 (38.7)	44 (57.1)	
Cirrhosis				.673
Cirrhosis-negative	143 (63.6)	50 (66.7)	46 (59.7)	
Cirrhosis-positive	82 (36.4)	25 (33.3)	31 (40.3)	
Arterial peritumoral enhancement				.091
Arterial peritumoral enhancement-negative	48 (21.3)	22 (14.7)	26 (33.8)	
Arterial peritumoral enhancement-positive	177 (78.7)	64 (85.3)	51 (66.2)	
*Tumor number*				.655
Solitary	175 (77.8)	62 (82.7)	60 (77.9)	
Multiple	50 (22.2)	13 (17.3)	17 (22.1)	
*Laboratory findings*^*#*^				
AST (IU/mL)	31.5 (25.0–48.0)	48.4 (31.9–62.8)	26.5 (20.6–39.3)	.191
ALT (IU/mL)	31.7 (27.0–40.9)	48.1 (31.7–48.1)	28.6 (17.7–41.6)	.131
RBC (10^12/L)	4.5 (4.2–4.7)	4.5 (4.2–4.6)	4.5 (4.1–4.8)	.500
Hemoglobin (g/L)	141 (130.7–148)	142.2 (130.0–142.2)	146.0 (130.0–153.0)	.458
Platelet (10^9/L)	149.5 (116.0–174.0)	137.5 (137.5–146.6)	137.0 (102.0–177.0)	.319
TBil (µmol/L)	14.1 (11.6–16.8)	19.9 (12.0–24.6)	12.1 (10.0–16.3)	.220
DBil (µmol/L)	4.3 (3.3–5.2)	4.3 (3.2–5.2)	3.7 (2.8–4.7)	.082
Total protein (g/L)	67.2 (64.1–70.4)	62.4 (62.4–65.7)	65.7 (61.8–69.0)	.185
Albumin (g/L)	39.5 (38.0–41.1)	38.3 (38.3–39.7)	39.4 (37.5–41.3)	.210
CRP (mg/L)	4.6 (3.4–6.3)	11.4 (4.9–11.4)	4.5 (2.5–8.3)	.457
*ALBI grade*				.432
1	118 (52.4)	35 (46.7)	44 (57.1)	
2 or 3	107 (47.6)	40 (53.3)	33 (42.9)	
*Tumor markers*^*#*^				
AFP (ng/mL)	239.1 (8.5–348.3)	237.9 (10.0–472.2)	6.5 (2.8–201.9)	.059
CEA (ng/mL)	1.7 (0.9–1.9)	1.7 (1.0–1.9)	1.3 (0.6–2.3)	.138
CA242 (U/mL)	5.1 (2.7–6.7)	4.9 (3.2–9.6)	4.2 (3.4–6.2)	.259
*MVI*				.197
MVI = 0	159 (70.7)	46 (61.3)	57 (74.0)	
MVI = 1	66 (29.3)	29 (38.7)	20 (26.0)	

Except where indicated, data are numbers of patients, with percentages in parentheses.

^#^Data are medians, with interquartile range in parentheses

*HBV* Hepatitis B virus, *HCV* Hepatitis C virus, *AST* Aspartate aminotransferase, *ALT* Alanine transferase, *RBC* Red blood cells, *TBil* Total bilirubin, *DBil* Direct bilirubin, *CRP* C-reactive protein, *ALBI grade* Albumin-Bilirubin (ALBI) grade, *AFP* Alpha-fetoprotein, *CEA* Carcinoembryonic, *CA242* Carbohydrate antigen 242, *MVI* Microvascular invasion

### Risk factors for MVI

In the training cohort, maximum tumor diameter, tumor margin, arterial peritumoral enhancement, tumor number, hemoglobin, total bilirubin, direct bilirubin, and AFP were predictive clinical and imaging variables for the presence of MVI in HCC patients (*p* < 0.05) (Table 2). Multiple logistic regression analysis identified tumor margin, direct bilirubin, maximum tumor diameter, and AFP as independent risk factors associated with the risk of MVI (Table 3). Based on ROC curve analysis, we determined the best threshold values for maximum tumor diameter, direct bilirubin, and AFP to be 50 mm, 2.7 µmol/L, and 360.7 ng/mL, respectively. Meanwhile, in conjunction with the upper limits of normal values for direct bilirubin and AFP (6.8 µmol/L and 10 ng/mL, respectively), we defined the threshold values for the three-way classification in the nomogram, as described below.

**Table 2:** Comparison between MVI = 0 and MVI = 1 in the training cohort

Characteristics	MVI = 0 (*n* = 159)	MVI = 1 (*n* = 66)	*P*-value
Age in years^#^	60 (53–65)	58 (50–63)	.128
*Sex*			.440
No. of men	130 (81.8)	51 (77.3)	
No. of women	29 (18.2)	15 (22.7)	
*Liver disease*			.434
HBV infection	141 (88.7)	61 (92.4)	
HCV infection	6 (3.8)	3 (4.5)	
Other	12 (7.5)	2 (3.0)	
Maximum diameter^#^	41 (26.5–63.5)	55 (34.3–88.5)	.002*
*Tumor margin*			< .001*
Non-smooth	65 (40.9)	44 (59.1)	
Smooth	94 (66.7)	22 (33.3)	
*Cirrhosis*			.916
Cirrhosis-negative	100 (62.9)	42 (63.6)	
Cirrhosis-positive	59 (37.1)	24 (36.4)	
*Arterial peritumoral enhancement*			.030*
Arterial peritumoral enhancement-negative	40 (25.2)	8 (12.1)	
Arterial peritumoral enhancement-positive	119 (74.8)	58 (87.9)	
*Tumor number*			.005*
*Solitary*	132 (83.0)	43 (65.2)	
Multiple	27 (17.0)	23 (34.8)	
*Laboratory findings*^#^			
AST (IU/mL)	30.6 (27–40.6)	31.9 (26.9–40.5)	.343
ALT (IU/mL)	33.4 (25.2–48.0)	30.6 (24.2–39.6)	.643
RBC (10^12/L)	4.5 (4.2–4.6)	4.5 (4.3–4.7)	.057
Hemoglobin (g/L)	139.5 (129.0–146.8)	142.0 (135.0–148.5)	.017*
Platelet (10^9/L)	149.7 (112.7–173.0)	149.5 (116.2–182.5)	.657
TBil (µmol/L)	14.0 (11.4–16.6)	14.6 (12.0–17.5)	.043*
DBil (µmol/L)	4.3 (3.2–5.2)	4.5 (3.5–5.5)	.040*
Total protein (g/L)	67.3 (64.1–70.7)	67.1 (64.2–70.0)	.597
Albumin (g/L)	39.8 (38.0–41.1)	38.8 (37.9–40.9)	.517
CRP (mg/L)	4.5 (3.3–5.6)	4.9 (3.5–10.9)	.199
*ALBI grade*			.110
1	89 (56.0)	29 (43.9)	
2 or 3	70 (44.0)	37 (56.1)	
*Tumor markers*^#^			
AFP (ng/mL)	181.3 (5.1–325.2)	325.2 (36.1–1000.0)	.002*
CEA (ng/mL)	1.8 (0.9–1.9)	1.5 (0.7–2.1)	.671
CA242 (U/mL)	5.6 (3.1–6.6)	3.9 (2.5–6.6)	.698

Except where indicated, data are numbers of patients, with percentages in parentheses

*HBV* Hepatitis B virus, *HCV* Hepatitis C virus, *AST* Aspartate aminotransferase, *ALT* Alanine transferase, *RBC* Red blood cells, *TBil* Total bilirubin, *DBil* direct bilirubin, *CRP* C-reactive protein, *ALBI grade* Albumin-Bilirubin (ALBI) grade, *AFP* Alpha-fetoprotein, *CEA* Carcinoembryonic, *CA242* Carbohydrate antigen 242, *MVI* Microvascular invasion

^#^Data are medians, with interquartile range in parentheses

**Table 3:** ROC analysis and multivariate analysis of MVI presence in the training cohort

Variables	Multivariate analysis			ROC analysis	
	*β*	OR	*P*-value	AUC	Cutoff
Maximum diameter	0.015	1.015 (1.006,1.025)	.002	0.639 (0.558,0.719)	50
Tumor margin	1.259	3.523 (1.806,6.873)	< .001	0.632 (0.563,0.701)	NA
Arterial peritumoral enhancement	NA	NA	.999	NA	NA
Tumor number	NA	NA	.081	NA	NA
Hemoglobin	NA	NA	.083	NA	NA
TBil	NA	NA	.581	NA	NA
DBil	0.043	1.044 (1.012,1.076)	.007	0.567 (0.485,0.65)	2.7
AFP	0.001	1.001 (1.000,1.002)	.032	0.629 (0.547,0.711)	360.7

*NA* Not applicable, *TBil* Total bilirubin, *DBil* Direct bilirubin, *AFP* Alpha-fetoprotein, *OR* Odds ratio, *ROC* Receiver operating characteristic, *AUC* Area under the curve

### Development and validation of the prediction nomogram

**Fig. 3:**
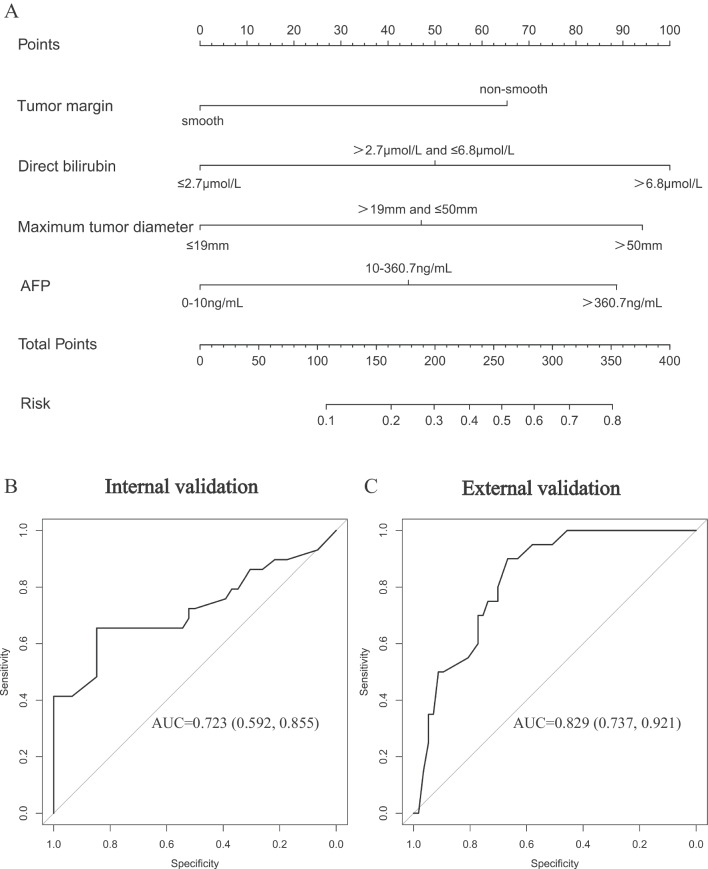
Developed nomogram presented with ROC. **A** The nomogram was established due to the training cohort, with tumor margin, direct bilirubin, maximum tumor diameter and AFP. **B** The ROC curve in the internal validation cohort. **C** The ROC curve in the external validation cohort. Abbreviations: AFP, alpha-fetoprotein; ROC, receiver operating characteristic; AUC, area under the curve

Using the outcomes of univariate and multivariate analyses, we developed a nomogram that included preoperative predictor variables for the presence of MVI in HCC patients. Figure 3A shows the score points for the nomogram, including maximum tumor diameter (0, ≤ 19 mm; 1, > 19 mm and ≤ 50 mm; 2, > 50 mm), tumor margin (0, smooth; 1, non-smooth), direct bilirubin (0, ≤ 2.7 µmol/L; 1, > 2.7 µmol/L and ≤ 6.8 µmol/L; 2, > 6.8 µmol/L), and AFP (0, 0–10 ng/mL; 1, 10–360.7 ng/mL; 2, > 360.7 ng/mL). The formula for the weighted value is: Y = 0.693 × [maximum tumor diameter] + 0.961 × [tumor margin + 0.652 × [direct bilirubin] + 0.736 × [AFP] – 5.859. Using thresholds determined in the training cohort, the internal and external validation cohort had a sensitivity range of 67.9–70%, specificity range of 68.1–75.4%, and accuracy range of 68–74% in diagnosing MVI (Table 4). We plotted ROC curves, and our nomogram model exhibited good diagnostic performance with AUC = 0.723 and AUC = 0.829 in the internal and external validation cohort, respectively (Fig. 3B and 3C).

**Table 4:** Diagnostic performance of models for predicting MVI in validation cohorts

Parameter	Internal validation cohort	External validation cohort
Sensitivity (%)	67.9 (19/28) [49.4, 86.3]	70.0 (14/20) [48.0, 92.0]
Specificity (%)	68.1 (32/47) [54.3, 81.9]	75.4 (43/57) [63.9, 87.0]
Accuracy (%)	68.0 (51/75) [57.2, 78.8]	74.0 (57/77) [64.0, 84.0]

Data in parenthesis are numerator/denominator and data in brackets are 95% confidence interval

## Discussion

This study developed and validated a nomogram model for preoperative prediction of MVI in HCC patients. Tumor margin, direct bilirubin, maximum tumor diameter, and AFP integrated into the nomogram model were confirmed as independent predictors of MVI risk. Subsequently, ROC curves for the internal and external validation cohorts demonstrated the excellent diagnostic performance of our model.

MVI is an independent risk factor for HCC prognosis and indicates a higher likelihood of tumor aggressiveness and metastasis [18, 19]. The factors included in our model have also been reported associated with MVI in other studies [16, 17, 20–22], demonstrating the reliability and reproducibility of our model. He YZ et al. developed the MVI preoperative scoring system based on tumor margin, maximum tumor diameter, AST, circulating tumor cells, Des-*γ*-carboxy-prothrombin (DCP), and AFP, which demonstrated better diagnostic performance than our model, with an AUC of 0.922 [22]. Xu X et al. developed a nomogram including eight independent risk factors based on radiomic analysis of contrast-enhanced CT [21]. However, these radiological features need to be got with specific computers and software, which is not practical for the surgeon's clinical work. Thus, perhaps the greatest strength of our model is its ease of application by clinicians. We used the image features (tumor size and tumor margins) obtained by subjective evaluation on contrast-enhanced CT to construct the model. This allows the radiologists or surgeons to quickly record the image features in clinical practice without taking too much time. Moreover, the fact is that many of the models that exist have not been externally validated. And in predictive research, external validation is necessary as the overfitting problem (prediction models tend to perform better on data on which the model was developed than on new data). Although a recent prospective study showed that the use of a ‘hepatocyte-specific’ contrast agent, Gadoxetic acid (Gd-EOB-DTPA), in magnetic resonance significantly improved the sensitivity of diagnosing small hepatocellular carcinoma (1–2 cm) in patients with cirrhosis, this examination is two to three times more expensive than contrast-enhanced CT [23]. We have developed a user-friendly, non-invasive, and externally validated predictive model. In addition to considering diagnostic performance, cost and applicability are also important. The independent predictors in our model can all be got through an outpatient examination.

Similar to tumor size and peripheral enhancement, non-smooth tumor margins are also an essential marker for assessing malignancy, as this often means the tumor has invaded the liver more [24]. In a systematic review and meta-analysis conducted by Song L et al., MVI was significantly associated with non-smooth tumor margins (DOR = 4.62 [2.73, 7.81]) with an AUC of 0.72 [0.60, 0.77] [20]. The maximum tumor diameter greater than or equal to 59 mm is an independent risk factor in the MVI preoperative scoring system developed by He YZ et al. [22]. Some recent studies have confirmed that the larger the diameter, the greater the likelihood of MVI [16, 17]. Unconjugated bilirubin is transported through the bloodstream to the liver and forms direct bilirubin. Elevated bilirubin levels indicate underlying liver function abnormalities, and changes in total bilirubin are significantly associated with the progression of HCC [25]. Chan AWH et al. showed that high albumin-bilirubin (ALBI) grade is a crucial parameter for early recurrence of HCC [26]. Mao S et al. first identified total bilirubin as a significant predictor of MVI when integrating tumor diameter and AFP [17]. In addition, total bilirubin has been reported as a significant risk factor in predictive models for OS and disease-free survival in patients with early-stage HCC [27, 28]. For the first time, we have identified direct bilirubin as an independent risk factor for MVI, but the interrelationship between the two is still not well understood. AFP ≥ 400 ng/mL is highly suggestive of HCC after excluding pregnancy, chronic or active liver disease, tumors of embryonic origin in the gonads, and tumors of the gastrointestinal tract, according to the HCC management guidelines. However, it has been shown that AFP ≥ 158 ng/mL (AUC = 0.752) is an independent risk factor for MVI [22], suggesting that high levels of AFP can also respond to tumor cell invasion [29].

We found that close to 30.5% of the 377 patients included had a postoperative pathological diagnosis of MVI. Although there is no recognized adjuvant therapy to reduce the recurrence of HCC after resection, patients at high risk of recurrence are potential candidates for clinical trials of adjuvant therapy [30]. Furthermore, it is possible to explore neoadjuvant therapy for patients at high risk of MVI. EASL also encouraged future clinical trials with new agents. At the same time, liver transplantation is the most effective treatment to prevent a recurrence. Partial hepatectomy for initially resectable HCC and salvage liver transplantation in transplantable tumor recurrence is one strategy [31]. These require the clinician to determine the patient's risk of tumor recurrence early and accurately, so preoperative assessment of the presence of MVI is necessary. We recommend that patients judged to be at high risk of MVI preoperatively by our model require greater care by pathologists in the postoperative assessment of pathological biopsies to reduce the rate of missed diagnoses. In addition, these patients should undergo a more prolonged and more intensive individualized surveillance program to detect possible tumor recurrence at an early stage.

However, there are limitations to this study that are worth noting. Firstly, this is also a retrospective study with selection bias despite internal and external validation to improve reliability. Secondly, we included patients with HCC due to HCV infection or other etiologies. However, the realities in China resulted in our inclusion of mostly HBV-related HCC patients, which led to some unavoidable bias. Thirdly, DCP was not measured in our study. DCP was not routinely tested preoperatively from 2017 to 2020 in our hospital. This is partly because the EASL guidelines stated that DCP is suboptimal in terms of cost-effectiveness for routine surveillance of early HCC [32]. At present, DCP is a routinely tested tumor marker and will definitely be included in future studies. Fourthly, our model has not been validated by prospective clinical trials. Furthermore, the accuracy of the pathological diagnosis of MVI depends mainly on the experience of pathologists. We share this model to raise awareness of risk factors for MVI in HCC and facilitate the conduct of multi-center prospective studies to further improve the diagnostic accuracy of the model.

## Conclusion

This study developed and validated a preoperative prediction model for MVI combining laboratory examinations and contrast-enhanced CT imaging features, which includes maximum tumor diameter (> 50 mm), tumor margin, direct bilirubin (> 2.7 µmol/L), and AFP (> 360.7 ng/mL). The model helps in the early diagnosis of MVI. When predicting HCC patients with a high risk of MVI, the surgeons may perform an anatomical or wide-margin hepatectomy on the patient.

## Data Availability

The data used in this study is not a public data. The datasets used in this study are available from the corresponding authors on reasonable requests.

## References

[1] Zhang S, Sun K, Zheng R, Zeng H, Wang S, Chen R (2021). Cancer incidence and mortality in China, 2015. J Nat Cancer Center.

[2] Kang KJ, Ahn KS (2017). Anatomical resection of hepatocellular carcinoma: a critical review of the procedure and its benefits on survival. World J Gastroenterol.

[3] Banerjee S, Wang DS, Kim HJ, Sirlin CB, Chan MG, Korn RL (2015). A computed tomography radiogenomic biomarker predicts microvascular invasion and clinical outcomes in hepatocellular carcinoma. Hepatology.

[4] Özgün G, Haberal Reyhan N, Özdemir BH, Haberal M (2017). Liver transplant for hepatocellular carcinoma: pathologic point of view. Exp Clin Transplant.

[5] Famularo S, Piardi T, Molfino S, Di Martino M, Ferrari C, Ielpo B (2021). Factors affecting local and intra hepatic distant recurrence after surgery for Hcc: an alternative perspective on microvascular invasion and satellitosis - a Western European multicentre study. J Gastrointest Surg.

[6] Chong HH, Yang L, Sheng RF, Yu YL, Wu DJ, Rao SX (2021). Multi-scale and multi-parametric radiomics of gadoxetate disodium-enhanced MRI predicts microvascular invasion and outcome in patients with solitary hepatocellular carcinoma ≤ 5 cm. Eur Radiol.

[7] Kluger MD, Salceda JA, Laurent A, Tayar C, Duvoux C, Decaens T (2015). Liver resection for hepatocellular carcinoma in 313 Western patients: tumor biology and underlying liver rather than tumor size drive prognosis. J Hepatol.

[8] Zhao H, Chen C, Gu S, Yan X, Jia W, Mao L (2017). Anatomical versus non-anatomical resection for solitary hepatocellular carcinoma without macroscopic vascular invasion: a propensity score matching analysis. J Gastroenterol Hepatol.

[9] Yang P, Si A, Yang J, Cheng Z, Wang K, Li J (2019). A wide-margin liver resection improves long-term outcomes for patients with HBV-related hepatocellular carcinoma with microvascular invasion. Surgery.

[10] Wang W, Guo Y, Zhong J, Wang Q, Wang X, Wei H (2021). The clinical significance of microvascular invasion in the surgical planning and postoperative sequential treatment in hepatocellular carcinoma. Sci Rep.

[11] Granito A, Forgione A, Marinelli S, Renzulli M, Ielasi L, Sansone V (2021). Experience with regorafenib in the treatment of hepatocellular carcinoma. Therap Adv Gastroenterol.

[12] Tovoli F, Negrini G, Benevento F, Faggiano C, Goio E, Granito A (2018). Systemic treatments for hepatocellular carcinoma: challenges and future perspectives. Hepat Oncol.

[13] Iasonos A, Schrag D, Raj GV, Panageas KS (2008). How to build and interpret a nomogram for cancer prognosis. J Clin Oncol.

[14] Zhang C, Zhao R, Chen F, Zhu Y, Chen L (2021). Preoperative prediction of microvascular invasion in non-metastatic hepatocellular carcinoma based on nomogram analysis. Transl Oncol.

[15] Wang L, Jin YX, Ji YZ, Mu Y, Zhang SC, Pan SY (2020). Development and validation of a prediction model for microvascular invasion in hepatocellular carcinoma. World J Gastroenterol.

[16] Zhou Q, Zhou C, Yin Y, Chen W, Liu C, Atyah M, et al Development and validation of a nomogram combining hematological and imaging features for preoperative prediction of microvascular invasion in hepatocellular carcinoma patients. Ann Transl Med 2021; 9(5):402. 10.21037/atm-20-4695. 10.21037/atm-20-4695 PMC8033313 33842623

[17] Mao S, Yu X, Yang Y, Shan Y, Mugaanyi J, Wu S (2021). Preoperative nomogram for microvascular invasion prediction based on clinical database in hepatocellular carcinoma. Sci Rep.

[18] Erstad DJ, Tanabe KK (2019). Prognostic and therapeutic implications of microvascular invasion in hepatocellular carcinoma. Ann Surg Oncol.

[19] Rodríguez-Perálvarez M, Luong TV, Andreana L, Meyer T, Dhillon AP, Burroughs AK (2013). A systematic review of microvascular invasion in hepatocellular carcinoma: diagnostic and prognostic variability. Ann Surg Oncol.

[20] Song L, Li J, Luo Y (2021). The importance of a nonsmooth tumor margin and incomplete tumor capsule in predicting HCC microvascular invasion on preoperative imaging examination: a systematic review and meta-analysis. Clin Imaging.

[21] Xu X, Zhang HL, Liu QP, Sun SW, Zhang J, Zhu FP (2019). Radiomic analysis of contrast-enhanced CT predicts microvascular invasion and outcome in hepatocellular carcinoma. J Hepatol.

[22] He YZ, He K, Huang RQ, Wang ZL, Ye SW, Liu LW (2020). Preoperative evaluation and prediction of clinical scores for hepatocellular carcinoma microvascular invasion: a single-center retrospective analysis. Ann Hepatol.

[23] Granito A, Galassi M, Piscaglia F, Romanini L, Lucidi V, Renzulli M (2013). Impact of gadoxetic acid (Gd-EOB-DTPA)-enhanced magnetic resonance on the non-invasive diagnosis of small hepatocellular carcinoma: a prospective study. Aliment Pharmacol Ther.

[24] Hussain HK, Barr DC, Wald C (2014). Imaging techniques for the diagnosis of hepatocellular carcinoma and the evaluation of response to treatment. Semin Liver Dis.

[25] Jiang N, Song X, Peng YM, Wang WN, Song Z (2020). Association of disease condition with changes in intestinal flora, and plasma endotoxin and vascular endothelial growth factor levels in patients with liver cancer. Eur Rev Med Pharmacol Sci.

[26] Chan AWH, Zhong J, Berhane S, Toyoda H, Cucchetti A, Shi K (2018). Development of pre and post-operative models to predict early recurrence of hepatocellular carcinoma after surgical resection. J Hepatol.

[27] Kong W, Gao M, Jin Y, Huang W, Huang Z, Xie Z Prognostic model of patients with liver cancer based on tumor stem cell content and immune process. Aging Albany NY 2020;12(16):16555–16578. 10.18632/aging.103832. 10.18632/aging.103832 PMC7485734 32852285

[28] Li Y, Zhang Y, Fang Q, Zhang X, Hou P, Wu H (2021). Radiomics analysis of [18F]FDG PET/CT for microvascular invasion and prognosis prediction in very-early- and early-stage hepatocellular carcinoma. Eur J Nucl Med Mol Imaging.

[29] Ryu T, Takami Y, Wada Y, Tateishi M, Hara T, Yoshitomi M (2019). A clinical scoring system for predicting microvascular invasion in patients with hepatocellular carcinoma within the milan criteria. J Gastrointest Surg.

[30] Heimbach JK, Kulik LM, Finn RS, Sirlin CB, Abecassis MM, Roberts LR (2018). AASLD guidelines for the treatment of hepatocellular carcinoma. Hepatology.

[31] de Haas RJ, Lim C, Bhangui P, Salloum C, Compagnon P, Feray C (2018). Curative salvage liver transplantation in patients with cirrhosis and hepatocellular carcinoma: an intention-to-treat analysis. Hepatology.

[32] European association for the study of the liver. Electronic address: easloffice@easloffice.eu; European Association for the study of the liver. EASL clinical practice guidelines: management of hepatocellular carcinoma. J Hepatol. 2018;69(1):182–236. 10.1016/j.jhep.2018.03.019. 10.1016/j.jhep.2018.03.019 29628281

